# No Detection of XMRV in Blood Samples and Tissue Sections from Prostate Cancer Patients in Northern Europe

**DOI:** 10.1371/journal.pone.0025592

**Published:** 2011-10-12

**Authors:** Kristin Stieler, Sarah Schindler, Thorsten Schlomm, Oliver Hohn, Norbert Bannert, Ronald Simon, Sarah Minner, Michael Schindler, Nicole Fischer

**Affiliations:** 1 Institute for Medical Microbiology and Virology, University Medical Center Hamburg-Eppendorf, Hamburg, Germany; 2 Heinrich-Pette Institute, Leibniz Institute for Experimental Virology, Hamburg, Germany; 3 Martini-Clinic, Prostate Cancer Center, University Medical Center Hamburg-Eppendorf, Hamburg, Germany; 4 Robert Koch Institute, Center for HIV and Retrovirology, Berlin, Germany; 5 Institute of Pathology, University Medical Center Hamburg-Eppendorf, Hamburg, Germany; University of Kentucky College of Medicine, United States of America

## Abstract

**Background:**

We recently published the rare detection of xenotropic murine leukemia virus-related virus (XMRV) (1/105) in prostate cancer (PCA) tissue of patients in Northern Europe by PCR. The controversial discussion about the virus being detected in PCA tissue, blood samples from patients suffering from chronic fatigue syndrome (CFS), as well as from a significant number of healthy controls prompted us to deepen our studies about detection of XMRV infection applying different detection methods (PCR, cocultivation and immunohistochemistry [IHC]).

**Methodology/Principal Findings:**

Peripheral blood mononuclear cells (PBMCs) from 92 PCA and 7 healthy controls were isolated, PHA activated and cocultivated with LNCaP cells for up to 8 weeks. Supernatant of these cells was applied to a reporter cell line, DERSE-iGFP. Furthermore, the PBMCs and cocultivated LNCaP cells were tested for the presence of XMRV by PCR as well as Western Blot analysis. While all PCR amplifications and Western Blot analyses were negative for signs of XMRV infection, DERSE-iGFP cells displayed isolated GFP positive cells in three cases. In all three cases XMRV presence could not be confirmed by PCR technology. In addition, we performed XMRV specific IHC on PCA tissue sections. Whole tissue sections (n = 20), as well as tissue microarrays (TMA) including 50 benign prostate hyperplasia (BPH), 50 low grade and 50 high grade PCA sections and TMAs including breast cancer, colon cancer and normal tissues were stained with two XMRV specific antisera. XMRV protein expression was not detected in any cancer sections included. One BPH tissue displayed XMRV specific protein expression in random isolated basal cells.

**Conclusion:**

We were unable to conclusively detect XMRV in the blood from PCA patients or from healthy controls and there is no conclusive evidence of XMRV protein expression in PCA, breast cancer and colon cancer tissue sections tested by IHC staining.

## Introduction

Currently, the detection of Xenotropic Murine Leukaemia Virus related Retrovirus (XMRV) in human bio specimens is controversially discussed ranging from XMRV being associated with two major human diseases, chronic fatigue syndrome (CFS) [1], [2] and prostate cancer (PCA) [3], [4] to being a men generated laboratory contaminant due to xenograft passaging through mice [5]–[18].

In 2006, XMRV has been identified in prostate tissue from patients with familiar prostate cancer (PCA) carrying a homozygous mutation within the RNaseL gene (R462Q) [19]. The association between XMRV and PCA was severely strengthened by studies demonstrating XMRV protein expression as well as the presence of XMRV sequences in up to 26% of all PCA cases [3], [4], [20]. XMRV protein expression was predominantly seen in malignant epithelium suggesting a more direct role in tumorigenesis. However, there are multiple studies only rarely or completely failing to detect XMRV in prostate cancer samples using PCR or IHC methods [3], [4], [9], [21]–[26]. We recently detected XMRV at low frequency (1%) in sporadic PCA samples from Northern Europe using PCR amplification methods and RNA isolated from fresh frozen tissue specimens [27]. Expression of XMRV protein as well as the presence of XMRV sequences in up to 26% of all analysed PCA samples was demonstrated in 2009 by applying immunohistochemistry (IHC) of whole mount PCA sections with an anti-XMRV specific antiserum [4], [20]. However, a recent report using Rauscher MLV gag antisera which also recognizes XMRV gag protein, did not confirm these findings [24]. The study by Schlaberg et al. prompted us to revisit the prevalence of XMRV in PCA samples by IHC since focal infections seen by IHC might be missed in PCR analysis. In addition, we evaluate the presence of XMRV protein expression in sections of other malignancies as well as normal tissue by IHC. By using the recently published anti-XMRV antiserum [4] as well as an XMRV gag specific antiserum we were unable to detect XMRV gag specific staining of cells in PCA or other cancerous tissue. However, one benign prostate hyperplasia (BPH) section clearly displayed positive stained cells using anti-XMRV gag k121 serum.

In 2009 XMRV was identified in up to 68% of PBMC (peripheral blood mononuclear cells) samples from patients with chronic fatigue syndrome and 3–4% of the control cohort showed signs of XMRV infection [2]. PCR data were strengthened by cell dependent as well as cell free transmission of the virus from blood samples of CFS patients to indicator cells. However, several subsequent studies by other labs failed to confirm the PCR data and no virus transmission experiments have been reproduced to date [6], [9], [10], [11], [13], [15], [17], [18], [28], [29], [30], [31]. Recently, blood samples from CFS patients previously reported to contain XMRV sequences were retested, however were identified as XMRV negative by PCR amplification strategies and serology methods [12], [32].

Earlier this year, while this study was in progress, several publications addressed the risk of contaminations by traces of mouse DNA (paraffin sections, cell lines or other sources) [7], [13], [15] and the risk of false positive PCR products by some commercial amplification kits [17], [33]. In addition, Hue and colleagues argue that due to the lack of sequence variability of XMRV gene fragments in patient isolates compared to sequence variability identified in a XMRV positive cell line 22Rv1, XMRV might be a laboratory contaminant rather than a true exogenous human virus [11]. A strong indication that XMRV is a virus circulating in the human population is the identification of viral integration sites in the host genome [34]. However, more recent findings demonstrate that two integration sites published earlier are identical to XMRV integration sites in an in vitro infected cell line DU145 [35]. Furthermore, Paprotka and colleagues provide evidence that XMRV derived from two mouse endogenous pre-viruses which underwent retroviral recombination in cell culture thereby suggesting that all XMRV sequences reported to date did most likely originate from this cell culture event [14]. In the presented study we addressed the detection of XMRV and related MLV sequences in peripheral blood cells of prostate cancer patients and healthy controls motivated by the detection of XMRV in blood cells of 3–4% of healthy controls [2] and our hypothesis that XMRV replication could be activated due to immunosuppression accompanying PCA and subsequently detectable in the blood of patients. A total of 100 blood samples were included in our study. PBMCs were isolated, stimulated and subsequently used for genomic DNA isolation or cocultivation experiments following published protocols [1], [2]. Furthermore, protein extracts from activated PBMCs were generated and analysed for XMRV protein expression. We show that PBMCs in general can be in vitro infected with XMRV, resulting in 1–2% infected cells which can be easily monitored by PCR or protein expression analyses thereby confirming recently published results [10]. Although viral genomes are highly edited due to Apobec restriction, supernatant from XMRV infected PBMCs efficiently infects a reporter cell line, DERSE-iGFP. This cell line (generated by Vineet N. KewalRamani, National Cancer Institute, Frederick, USA) expresses a GFP reporter which is activated by reverse transcriptase expression. Although the sensitivity of all techniques used in our study is fairly high, no XMRV sequences or XMRV specific protein expression was detected in activated PBMCs. Interestingly, we detected in supernatant from 3/67 activated PBMCs and 2/67 cocultivation experiments of PBMCs with LNCaP cells, RT activity resulting in GFP positive DERSE-iGFP cells, however, we were unable to unambiguously proof that these PBMCs have been infected with XMRV, other sources of RT activity can not be excluded.

## Materials and Methods

### Ethics statement

The study was approved by the Ethics Committee of the Federal State Hamburg (no. OB-052-04).

### Study population and specimen collection. Study population and specimen collection

Blood samples of 92 prostate cancer patients (age 44–77) were collected one day prior radical prostatectomy. Clinical data are summarized in Table 1. Additionally, blood samples from 7 men (age 30–44) without any evidence of PCA were included in the study. All patients gave written informed consent for the scientific use of blood samples; EDTA-blood from patients and healthy controls were processed by density gradient centrifugation using Ficoll (Biocoll, Biochrom L6715). Primary blood mononuclear cells (PBMCs) were separated and cultivated as described below.

**Table 1:** Summary of clinical data.

Patients PBMCs	n (%) 92
Age at surgery	
mean (years)	63
median (years)	63
range (years)	44–77
Gleason	
≤3+3	7 (7.6)
3+4	69 (75)
4+3	14 (15.2)
≥4+4	2 (2.2)
T stage	
pT2a	7 (7.6)
pT2c	57 (62)
pT3a	20 (21.7)
pT3b	8 (8.7)
N staus	
N0	69 (75)
N1	4 (4.3)
Nx	19 (20.7)

### Cell lines

The human prostate cancer cell line LNCaP (ATCC #CRL-1740), LNCaP DERSE-iGFP (kindly provided by Vineet N. KewalRamani, National Cancer Institute, Frederick, USA) and the XMRV positive human prostate cancer cell line 22Rv1 (ATCC #CRL-2505) were grown in RPMI 1640 (Gibco) supplemented with 10% FCS, 5% Penicillin/Streptomycin and L-glutamine. Chronically infected LNCaP cells (XMRV) were generated by transfection of proviral XMRV VP62 DNA as published previously [36] and maintained for several weeks. PBMC were isolated from 10 ml EDTA blood and cultured in RPMI 1640 (Gibco) similar to established prostate cancer cell lines but additionally supplemented with PHA (5 µg/ml, Thermo Fisher Scientific) and rhIL-2 (180 IU/ml, R&D Systems).

### Cocultivation experiments

1ml cell suspension containing 1×10^6^–3×10^6^ PBMCs activated for 7 days was added to 2×10^5^ LNCaP cells maintained in 2ml RPMI containing 8 µg/ml polybrene in 6-well plates. Plates were centrifuged for 30 min at 37°C and 800 × g. PBMCs were removed 24h later. LNCaP cells were cultured for 6–8 weeks. Cells were split when reaching 100% confluence. Supernatants were taken after 6 and 8 weeks and applied to DERSE-iGFP cells (see below).

For positive controls human PBMC were infected with XMRV-containing supernatant from LNCaP XMRV cells. Indicated amount of virus containing supernatant from XMRV producing cells (at least 80% confluence) was sterile filtered and added to 3×10^6^ PBMCs pre-activated for two days. Plates were centrifuged for 30 min at 37°C and 800 × g. XMRV containing supernatant was removed the next day by pelleting cells at 200 × g, washing them with 10 ml PBS (Gibco) and disseminating after an additional centrifugation step in a new 6-well plate in 2 ml RPMI containing PHA and rhIL-2. PBMCs were cultivated for 7 days before analyzing supernatant, co-cultivation, nucleic acid and protein extraction.

### Infection using replication competent XMRV

XMRV VP62 proviral DNA was transfected into LNCaP cells to produce virus containing supernatant as described earlier [34], [36].

### PCR

Genomic DNA was extracted from PBMCs using Qiagen QIAamp mini kit and stored at 4°C. Nucleic acid concentrations were determined using a Nanodrop (Peqlab). Different nested PCRs targeting gag and env sequences were performed as recently published [1], [3], [19], using 650 ng template DNA per reaction. Gag outside primer: 419F 5′- ATCAGTTAACCTACCCGAGTCGGAC-3′, 1154R 5′-GCCGCCTCTTCTTCATTGTTCTC-3′; inside primer: NP116F 5′-CATGGGACAGACCGTAACTACC-3′and NP117R 5′-GCAGATCGGGACGGAGGTTG-3′. To determine the sensitivity of the Gag PCR originally published by Urisman et al. the following primers were applied: GAG OF 5′-CGCGTCTGATTTGTTTTGTT-3′, GAG OR 5′- CCGCCTCTTCTTCATTGTTC-3′, GAG IF 5′- TCTCGAGATCATGGGACAGA-3′ and GAG IR 5′- AGAGGGTAAGGGCAGGGTAA [19]. The env PCR was performed as recently published [3] using the following primer pairs F 5′-ACCAGACTAAGAACTTAGAACCTCG-3′, R5′-AGCTGTTCAGTGATCACGGGATTAG-3′, IF 5′-GAACAGCATGGAAAGTCCAGCGTTC-3′ and IR 5′-CAGTGGATCGATACAGTCTTAGTCC-3′. The integrity of the DNA samples and the presence of putative inhibiters were controlled by amplifying GAPDH, F 5′- GAAGGTGAAGGTCGGAGTC-3′ and R 5′- GAAGATGG TGATGGGATTTC-3′.

### Western Blot

Cell lysates were generated using RIPA buffer containing 1% Triton-X 100 and protease inhibitor mix (Roche). Specific protein bands were detected by polyclonal Env antibody Rauscher 77S85 (gift of C. Stocking, Heinrich-Pette Institute, Hamburg, Germany), XMRV specific rabbit polyclonal Gag antiserum k121 and p30-Gag recognizing hybridoma supernatant from CRL-1912 cells (ATCC). Equal protein amounts per lane were ensured with anti-human actin antibody mAB 1501 (Chemicon) incubation. For the detection of XMRV particles in cell culture supernatants, sterile filtered culture medium of infected cells was ultracentrifuged 1 h, 110.000×g at 4°C (Beckman SW60Ti). The pellet of 11ml supernatant was resuspended in 10 µl PBS and analyzed by immunoblotting.

### Cell line paraffin sections and TMAs

1×10^7^ cells (LNCaP, LNCaP chronically infected with XMRV, 293T, 293T chronically infected with XMRV and mouse SC1 cells) were fixed for 20 h in 10% phosphate buffered formalin, embedded in agar and processed to paraffin wax [37].

A preexisting TMA containing prostate tissue (50 low grade PCA, 50 high grade PCA and 50 benign prostate hyperplasia (BPH)) was used for IHC.

### Immunhistochemistry

Slides with paraffin sections of prostate cancer patients were initially deparaffinized using xylene. For antigen retrieval sections were heated 4×2 min in a citrate buffer using a microwave (650W) and then cooled down to room temperature for 30 min. Blocking was performed for 30 min at RT with 10% swine serum in antibody dilution buffer (Dako). Afterwards endogenous biotin was blocked using Avidin/Biotin Kit (Dako). Primary antibody (diluted in antibody dilution buffer with 2% swine serum, anti-XMRV 1∶7500; XMRV anti-gag k121 1∶5000) was incubated for 2 h at room temperature in a humid chamber. Controls were either coated with the corresponding pre serum (same dilution) or only with antibody dilution buffer with 2% swine serum. The incubation with the secondary antibody – biotin/streptavidin labeled – was performed for 30 min at RT. For a later detection of bound antibodies labeled sections were coated with alkaline phosphatase solution (Dako, AK 5000) according to manufactures instructions. IHC staining solution containing levamisole to inhibit endogenous alkaline phosphatase was added to the slides for 15–20 min, while counterstaining was performed with Mayers hamin solutions. The anti-XMRV serum was kindly provided by Ila Singh (University of Utah, USA).

## Results

### XMRV protein expression in PCA tissue by IHC methods

In 2009, the finding of 23% of PCA sections positive for XMRV protein expression has been reported [4]. XMRV protein expression which in the majority of cases localized to the tumor epithelium strongly correlated with higher Gleason grades. Interestingly, the protein expression data did not correlate with PCR results. One putative explanation being few focal infected XMRV cells in the prostate which are hardly detectable by PCR using DNA from whole mount tissue sections as template. However, these findings were not confirmed by another study [24]. To contribute to the explanation of the discrepancies we screened whole PCA sections as well as TMAs using the recently published anti-XMRV serum [4] and a rabbit polyclonal anti-XMRV gag serum (gag k121).

**Figure 1:**
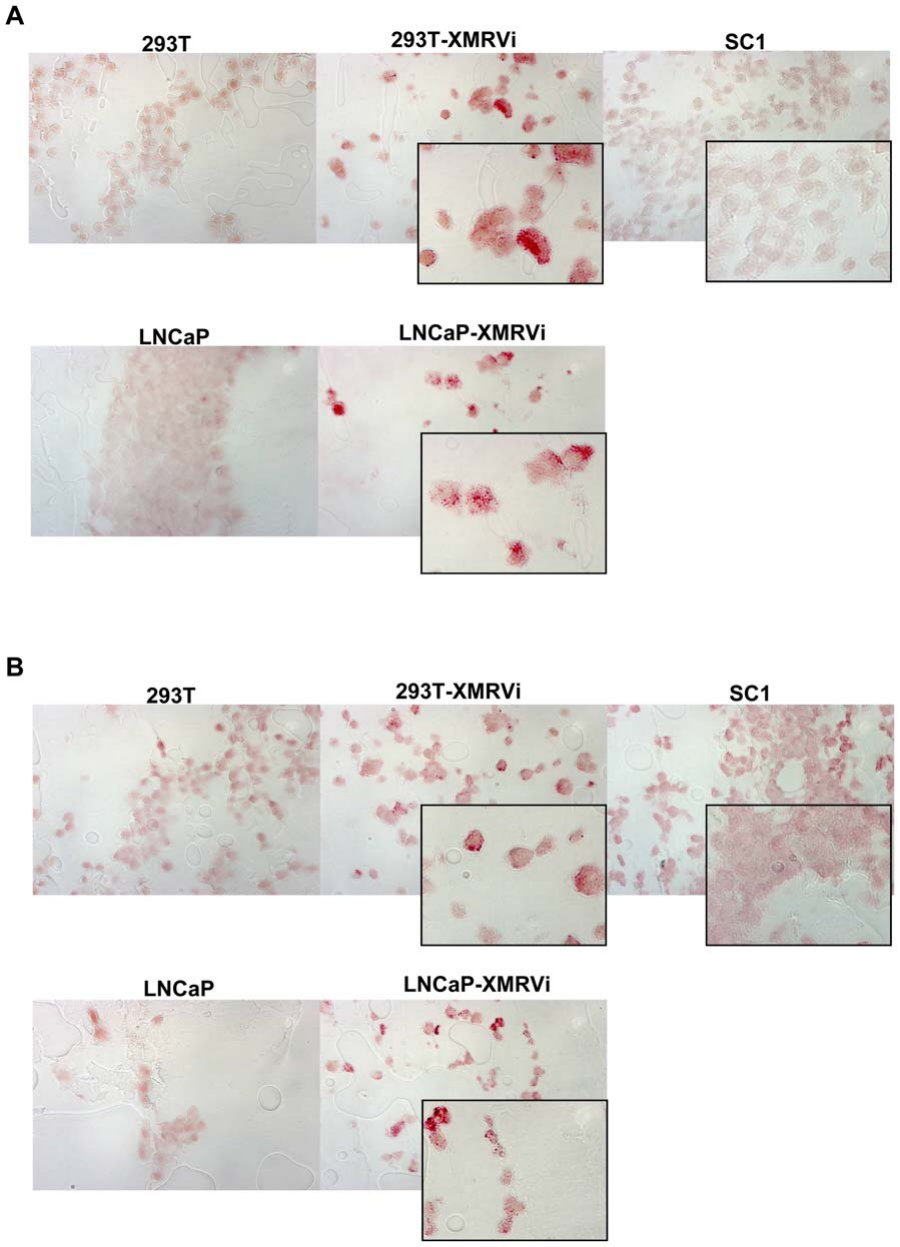
XMRV specific immunohistochemistry staining on cell line paraffin sections. Paraffin sections of cell line array containing XMRV infected cell lines as well as non infected cell lines were stained for XMRV protein expression using anti-XMRV serum (A) or anti-gag k121 polyclonal rabbit serum (B). Larger magnifications are displayed for XMRV infected cells as well as for a feral mouse cell line, SC1.

**Figure 2:**
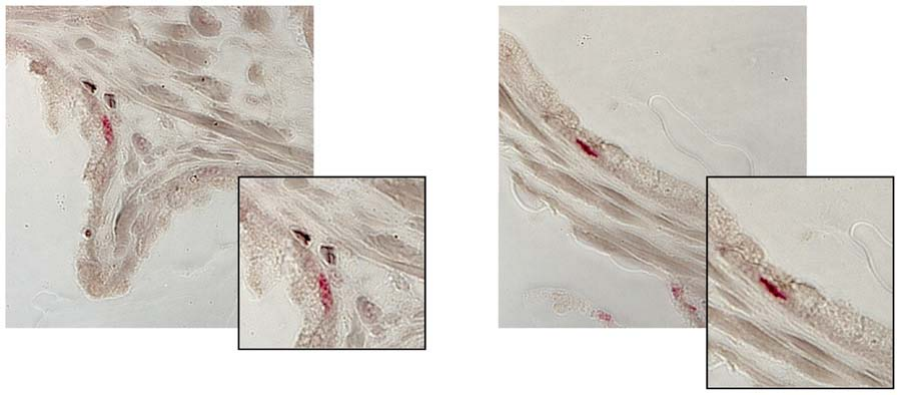
Immunohistochemistry staining using anti-gag k121 polyclonal rabbit serum on TMAs representing prostate cancer sections as well as benign prostate hyperplasia (BPH). In 1/50 BPH random positive stained cells were observed, which might be basal cells based on their localization in the prostate.

Both sera have been tested in Western Blot analyzes with gag k121 serum specifically recognizing xenotropic gag protein while displaying no cross reactivity with any cellular proteins. In contrast the anti-XMRV serum [4] also recognized cellular proteins in non infected human and mouse cell lines (supplementary Figure S1). We generated paraffin sections representing human cell lines 293T, LNCaP, both cell lines infected with XMRV and a mouse cell line SC1. Both antisera recognize XMRV protein expressing cells in paraffin sections showing granular staining of the cytoplasm (Figure 1). No staining of uninfected cells and no staining of SC1 mouse cells was detected. A total of 100 PCA (low grade and high grade PCA) and 50 BPH represented on a TMA as well as 10 large sections of prostate cancer (with high Gleason Score) were analyzed with gag k121 serum (Table 2). In addition a TMA containing breast, colon and prostate cancer as well as several normal tissues was tested for XMRV protein expression. Each IHC staining was controlled by including positive controls (paraffin sections of cell lines) and negative controls (without addition of first antibody) as well as higher dilutions of the first antibody. No staining of cancer sections was observed as well as the majority of control tissues was negative for gag k121 staining. Only one section of BPH displayed very few random basal cells staining positive with anti-gag k121 serum (Figure 2). None of the TMA was tested with the anti-XMRV serum since high background due to the TMA generation procedure has been observed.

**Table 2:** Summary of XMRV IHC on PCA sections and other common malignancies.

	α-XMRV (Schlaberg et al., PNAS 2009)	α-gag 121
**PCA TMA**	n.t.	0/50 high grade PCA
		0/50 low grade PCA
		**1**/50 BPH
**TMA** *	n.t.	0/114
**PCA tissue sections**	0/10 (high grade)	0/10 (high grade)

*: **Neoplasia**: Breast cancer, colon cancer; prostate cancer; **Normal tissue**: Adrenal gland, colon, endometrium, epididymis, heart, kidney, lung, pancreas, placenta, parotid gland, prostate, skin, spleen, stomach, striated muscle, thymus, tonsil, testis.

#### Activated PBMCs can be infected with XMRV, however XMRV replication is restricted in PBMCs

**Figure 3:**
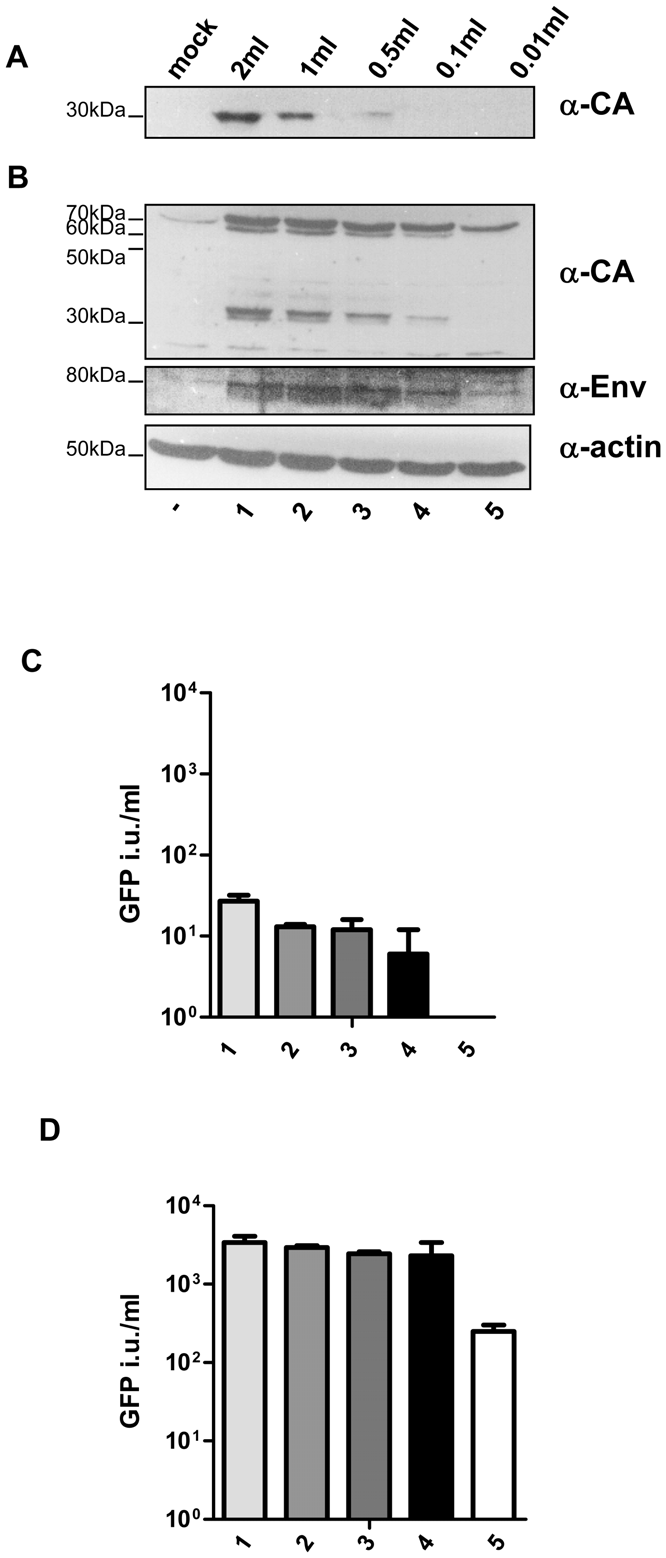
XMRV efficiently infects and replicates in peripheral blood mononuclear cells (PBMCs). PBMCs from two different donors were isolated, pooled, PHA stimulated and subsequently infected with the indicated amounts of XMRV containing supernatant (lane 1–5). Western Blot analysis of cell lysate from infected PBMCs was performed 7 d past infection (B). Supernatant of the infected PBMCs was enriched for virus particles by ultracentrifugation and stained for CA expression (A). (C) 500 µl of XMRV containing supernatant originated from PBMCs shown in A and B was used to infect DERSE-iGFP cells which were analysed for GFP expression 7 d past infection by FACS. Titers are indicated as GFP infectious units/ml. (D) Infection of DERSE-iGFP cells is 100fold increased by cocultivation of infected PBMCs (shown in (A)) with LNCaP cells for 7 d, SN of LNCaP cells was then applied to DERSE-iGFP cells, which were analysed by FACS 5 d p.i..

**Figure 4:**
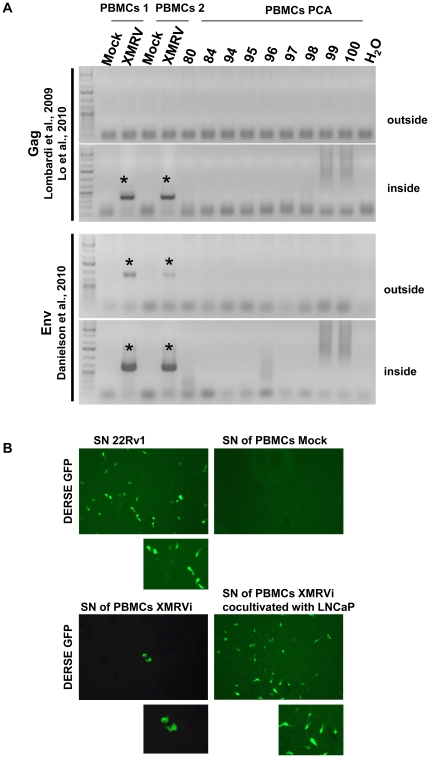
Detection of XMRV infection in PBMCs in vitro infected with XMRV by PCR (A), 650 ng genomic DNA isolated from PBMCs 7 d past infection were used as template. (B) DERSE-iGFP cells were infected with 500 µl supernatant from 22Rv1 cells, mock infected cells or LNCaP cells cocultured with XMRV infected PBMCs for 14 d. 72 h past infection DERSE-iGFP cells were monitored for GFP positive cells by microscopy.

Following the hypothesis published by Lombardi et al, that XMRV can be detected in PBMCs from up to 67% of CFS patients as well as in up to 4% of healthy controls [2] we intended to activate PBMCs from PCA patients and control patients and screen for XMRV infection applying different methods. We first established our XMRV detection methods on PBMCs which have been in vitro infected with viral supernatant containing VP62 XMRV. Proviral DNA was used to produce XMRV infectious supernatants in LNCaP cells which strongly support XMRV replication due to strong activation of the LTR as well as the lack of retroviral restriction factors Apobec 3G expression [36], [38]–[41]. PHA activated PBMCs were in vitro infected with the indicated amounts of viral supernatant (Figure 3) which were cultured in the presence of IL2 for another 7 d. Virus containing supernatant was then subjected to ultracentrifugation and viral pellets (Figure 3A) as well as cell lysate (Figure 3B) from the infected PBMCs were analyzed by Western Blotting ensuring the expression of XMRV specific proteins. Based on Western Blot experiments using chronically infected LNCaP cells diluted with the indicated cell number of uninfected 293T cells (Figure S2) we can estimate that approximately 1–2% of PBMCs are infected with XMRV. Only if we infect PBMCs with high viral titers we efficiently detected XMRV in the viral pellet after ultracentrifugation and Western Blot analysis (Figure 3A). Genomic DNA isolated from these in vitro XMRV infected PBMCs was positive for XMRV sequences by PCR using 650 ng genomic DNA and two different primer sets targeting gag and env (Figure 4A and Figure S3). Sensitivity of all PCR reactions is indicated in supplementary Figure S4 with all PCR detecting 1–10 infected cells in a background of 10^6^ uninfected cells.

#### Cocultivation of XMRV infected PBMCs with LNCaP cells significantly increases sensitivity of XMRV detection

DERSE-iGFP cells were exposed to filtered culture supernatant from XMRV infected PBMCs. 500 µl of supernatant was added to 5×10^4^ DERSE-iGFP cells which were scored for GFP expression 7 d p.i. by microscopy and FACS analysis (Figure 3C). In general, viral supernatant from PBMCs is infectious, however only very few GFP positive cells were detected. Interestingly, if we cocultivate the XMRV infected PBMCs with LNCaP cells for 5 d, harvest the supernatant and reinfect DERSE-iGFP cells with filtered supernatant, sensitivity of XMRV detection using DERSE-iGFP cells was 100fold increased Figure 3D and Figure 4B.

### PBMCs of PCA patients are negative for XMRV detection by PCR analysis

**Figure 5:**
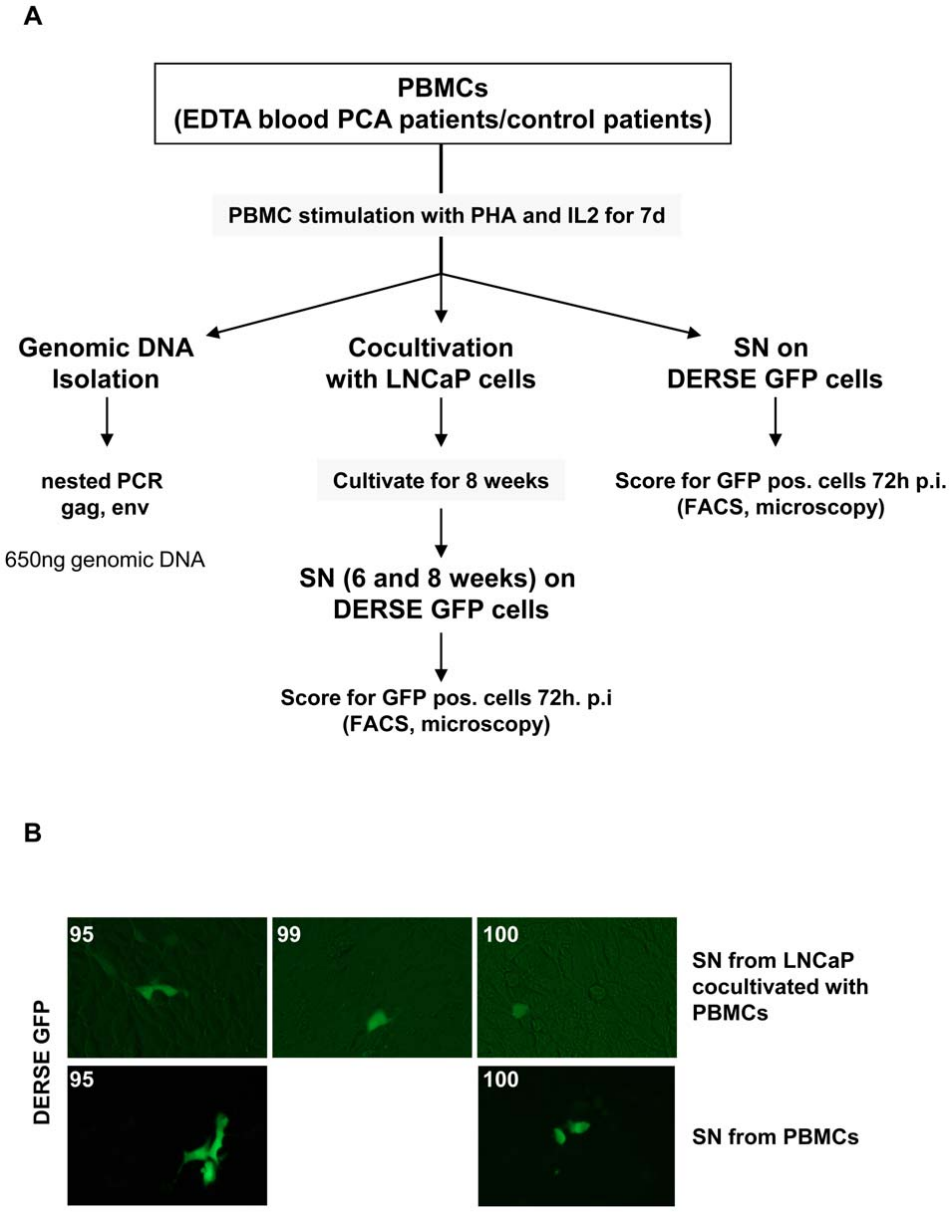
Detection of XMRV in PBMCs isolated from PCA patients and healthy controls. (A) Methods used to screen for XMRV in PBMCs of PCA patients and healthy controls. (B) DERSE-iGFP cells 72 h p.i. with SN from LNCaP cells cocultured for 8 weeks with patient derived PBMCs (upper panels). The lower panels display DERSE-iGFP cells 72 h p.i. with SN from patient derived PBMCs which were activated with PHA for 7d.

Using this approach we isolated PBMC from 92 PCA patients and 7 healthy volunteers by Ficoll gradient; isolated PBMCs were PHA activated and cultured in the presence of IL-2 for 7 d. PBMCs were subjected to different assays as outlines in Figure 5A: genomic DNA isolation followed by XMRV specific nested PCR applying two published XMRV PCR strategies [1], [3], [19]; cocultivation of activated PBMCs with LNCaP cells for 8 weeks with subsequent infection of DERSE-iGFP cells using supernatant 6 weeks and 8 weeks after cocultivation. Localization of the different primer sets used is shown in Figure S3 and sensitivity of the different XMRV PCRs is reflected in Figure S4. The integrity of the genomic DNA together with the absence of putative PCR inhibitors was ensured by GAPDH amplification (Figure S4). The culturing of PBMCs, DNA preparations and the PCR amplification were performed in laboratories of the Heinrich-Pette Institute where no other XMRV studies were performed. In addition, all nested PCRs to detect XMRV sequences using two different primer pairs targeting gag, both recently published, as well as an env PCR were run by two operators using 650 ng genomic DNA as template. All DNA samples were found to be consistently negative (Table 3). PCR reactions were routinely controlled for mouse contamination using primers directed against retrotransposons, intracisternal A particle (IAP), as recently published [15]. None of the PCR reactions was positive for mouse DNA sequences (data not shown).

**Table 3:** Summary of XMRV detection in activated PBMCs from PCA patients using nested PCR amplification from genomic DNA and coculture experiments on DERSE-iGFP cells.

	Nested PCR			Cell Culture	
	GAG (Urisman et al. PLoS Pathog. 2006)	GAG (Lo et al. PNAS 2010)	ENV (Danielson et al. JID 2010)	PBMCs cocultured with LNCaP^1^	SN from PBMCs on DERSE-iGFP cells^2^
**PCA Patients**	0/93	0/93	0/93	2/67	3*/10
**Healthy Controls**	0/7	0/7	0/7	n.t.	n.t.

1 Activated PBMCs were cocultured with LNCaP cells for 8 weeks. Supernatant of these LNCaP cells was applied to DERSE-iGFP cells.

2 Supernatant from activated PBMCs was applied to DERSE-iGFP cells without cocultivation with LNCaP cells.

* #99 and #100 derived from the same patient.

67 PBMC samples were cocultured with LNCaP cells for up to 8 weeks and SN of the LNCaP cells was applied to the reporter cell line DERSE-iGFP. This cell line carries a MLV vector, which leads to expression of a GFP reporter if reverse transcriptase is expressed. 72 h p.i. DERSE-iGFP cells were monitored for GFP expression by microscopy. Of 67 samples supernatant from PBMCs cocultured with LNCaP cells, two resulted in 2–3 GFP positive cells in 5×10^4^ cells (Figure 5B). We did not observe an increase of GFP positive cells over time indicating that there was no spread of viral infection. Interestingly the supernatant of the activated PBMCs from these two patients without cocultivation also resulted in 1–2 GFP positive DERSE-iGFP cells per well. In one case two independent PBMC isolations from the same patient were performed (#99 and #100) which both resulted in 1–2 GFP positive DERSE-iGFP cells. However, both isolations were performed at the same day by the same operator. PCR from LNCaP cells cocultured with PBMCs of these two patients did not result in detection of XMRV specific sequences as well as we were unable to culture and expand GFP positive DERSE-iGFP cells for subsequent analyses.

## Discussion

In this study we have examined the detection of XMRV in prostate cancer patients by studying different diagnostic bio specimens for the presence of XMRV or related MLV sequences. In particular, we analyzed PCA tissue specimens as well as tissue sections from other malignancies and normal tissues for XMRV protein expression by IHC. Furthermore, PBMCs from 92 PCA and 7 healthy controls were screened for the presence of XMRV sequences and recovery of infectious virus. PBMCs were PHA activated, cocultured for up to 8 weeks and XMRV presence was examined by either nested PCR targeting two different XMRV regions, Western Blot analyzes using different anti-XMRV antibodies or infection of DERSE-iGFP cells applying supernatant from activated PBMCs or supernatant from LNCaP cells cocultured with PBMCs for up to 8 weeks.

We were unable to conclusively show that XMRV sequences can be detected in activated PBMCs of PCA patients although in two patients GFP positive DERSE-iGFP cells were detected. In both cases subsequent PCR analyses of activated PBMCs as well as cocultured LNCaP cells were negative for XMRV sequences as well as we did not find XMRV protein expression in PCA sections of one of these patients.

We previously published that XMRV sequences are only rarely detected in Germany using cDNA generated from PCA tissue RNA amplified by PCR [27]. Similar results for a study in the US have been recently published by Switzer et al., [26]. However, there are multiple studies not identifying any XMRV sequences in PCA tissue as well as there are studies with higher prevalence of XMRV in PCA [3], [9], [21]–[24], [31], [42]. Considering the possibility of focal XMRV infection in the prostate which might be missed by PCR amplification due to only a minority of cells infected we established IHC staining using the published anti-XMRV serum and an XMRV specific anti-gag serum. We failed to detect XMRV protein expression in PCA tissue, breast cancer or colon cancer tissue as well as most control tissue (including 10 sections each: adrenal gland, colon, endometrium, epididymis, heart, kidney, lung, pancreas, placenta, parotid gland, spleen, stomach, striated muscle, thymus, tonsil, and testis) did not show any positive staining for gag k121 serum. Interestingly, using the anti-gag k121 serum we detected 1/50 BPH sections positive for XMRV protein expression. Protein expression was identified in a few isolated basal cells in the prostate epithelium. Basal cells are absent in PCA, supporting the fact that XMRV most likely is not directly involved in PCA development. The small number of whole mount tissue sections examined could account for the discrepancy between our findings and earlier findings by Schlaberg et al. [4]. We only stained ten whole mount tissue sections with both antisera, the anti-XMRV serum [4] was not used on TMA sections due to high background staining. Aloia et al. and Sakuma et al. both discuss a cross reactivity of anti-XMRV serum with human protein antigens resulting in IHC positive staining in PCA sections [16], [24]. We detect some cross reactivity with the published anti-XMRV serum on Western Blots analyzing cell lysates from infected and non infected cells, however there was no background observed on paraffin sections of cell lines or on whole sections of PCA tissue using serum at the indicated dilutions. Negative IHC staining does not exclude the possibility of few cells carrying XMRV proviral sequences which we might miss by PCR amplification. We did not apply DNA FISH technology to detect XMRV proviral integration in human tissue. Evaluation of FISH positive signal in 0.1% or less of the cells especially if only one viral copy per cell is to be expected, is highly error prone.

Recently, Lombardi et al. reported detection and transmission of infectious XMRV from PBMCs or plasma of patients with CFS by coculturing with LNCaP cells [2]. Interestingly, 3-4% of PBMCs isolated from control patients were identified to be positive for XMRV infectious virus resulting in the general concern about the safety of blood products. Several subsequent studies motivated by these results were unable to confirm these original findings. Reasons for the discordance are unclear and are currently investigated. While the majority of studies focussed on PCR techniques as well as detection of XMRV specific antibodies only one study included cocultivation of activated PBMCs from CFS patients with LNCaP cells [10] and a more recent study tested the transmission of XMRV from plasma (derived from CFS patients) to LNCaP cells [43]. Both studies did not detect XMRV in any of the samples tested. Focusing on the possibility that XMRV is a bystander virus reactivated in prostate cancer patients together with the finding that XMRV can be detected in PBMCs of patients [2] we searched for signs of XMRV infection in blood cells of PCA patients applying PCR technology and cocultivation of activated PBMCs with indicator cells. To our knowledge the current study is the first analyzing the presence of XMRV in blood samples from PCA patients in general and from a larger number of PBMCs (n = 92) tested by labor intensive coculturing of activated PBMCs with LNCaP cells for up to 8 weeks. A previous report by Hohn et al. also used cocultivation of activated PBMCs with subsequent genomic DNA isolation and XMRV specific amplification. Here we cocultivated activated PBMCs with LNCaP cells for up to 8 weeks (which increases sensitivity up to 100fold) and tested supernatant of these LNCaP cells for XMRV release by infection of DERSE-iGFP cells and subsequent FACS analysis or microscopy study.

In two patients we identified isolated GFP positive DERSE-iGFP cells when applying supernatant of activated PBMCs after 7 d as well as from the supernatant of LNCaP cells cocultivated for 8weeks with PBMCs. In all cases only very few positive cells were detected which could not be subcultivated to achieve significant cell numbers for subsequent experiments.

Taken together our data generated by analyzing different bio specimen, in particular tissue sections and PBMCs, for signs of XMRV infection do not support the association of XMRV with prostate cancer. Since we did not apply FISH technology to detect proviral integration we cannot exclude that few cell might show XMRV integration. However, the question of XMRV existence is different from the question of disease association. Our data are in concordance with recently published results demonstrating that XMRV can infect PBMCs in vitro [10], [44]. We find that 1–2% of PBMCs are infected when high amounts of viral titers are used for in vitro infection. These PBMCs release XMRV, however less viral particles are released compared to LNCaP cells and the virus is highly edited. Nevertheless, XMRV released from PBMCs is able to efficiently infect cells. Although we observed by two different experiments that DERSE-iGFP cells after incubation with supernatant from activated PBMCs express GFP in a few cells, we were unable to conclusively show that XMRV can be reactivated from PBMCs and infect an indicator cells line: no PCR detection of XMRV was achieved as well as the ultimate proof, cloning of integration sites from patients, is impossible from this material. At no time did we observe spontaneous GFP expression of DERSE-iGFP cells or GFP expression due to exogenous contamination of our cell culture, still contamination can not be experimentally ruled out.

In summary, we applied multiple methods to detect XMRV in bio specimen of prostate cancer patients; the results of our study do not support an association of XMRV and prostate cancer.

## Supporting Information

Figure S1 Western Blot analysis of XMRV negative (293T; LNCaP), XMRV positive human cell lines (22Rv1), chronically infected human cell lines (293T-XMRV; LNCaP-XMRV) as well as mouse cell lines (inbred NIH3T3 and feral mouse cells SC1) using rabbit polyclonal α-gag k121 serum (A) or rabbit polyclonal α-XMRV serum [4] (B) for detection.
(TIF)

Figure S2 Western Blot analysis of diluting amounts of chronically XMRV infected LNCaP cells mixed with non infected 293T cells. 25 µg total protein lysate was loaded per lane. Blots were immunoblotted using goat-anti env serum and rabbit-anti gag k121 serum. To ensure equal protein amounts loaded per lane the blot was reprobed with anti-actin monoclonal antibody.
(TIF)

Figure S3 XMRV VP62 Gag sequence 407-1160 (GI:89889045). Primers are indicated as arrows, GAG-O/I dark grey, 419F/1154R and NP116/NP117 light grey. Sequence variability between XMRV and MLV related sequences located in the indicated primer sequences are labeled with a star (*).
(TIF)

Figure S4 Genomic DNA was isolated from 1×10^6^ cells (indicated number of chronically XMRV infected LNCaP cells mixed with non infected 293T cells in 10 fold dilutions of infected cells in non infected cells). Nested PCR was performed using the oligos GAG-O and GAG-I [19], 419F/1154R and NP116/NP117 [1] as well as env primers 5604F/6491R and 5742F/6394R [3]. The highest dilution still showing XMRV specific amplification products in labelled with an *.
(TIF)

## References

[1] Lo SC, Pripuzova N, Li B, Komaroff AL, Hung GC (2010). Detection of MLV-related virus gene sequences in blood of patients with chronic fatigue syndrome and healthy blood donors.. Proc Natl Acad Sci U S A.

[2] Lombardi VC, Ruscetti FW, Das Gupta J, Pfost MA, Hagen KS (2009). Detection of an infectious retrovirus, XMRV, in blood cells of patients with chronic fatigue syndrome.. Science.

[3] Danielson BP, Ayala GE, Kimata JT (2010). Detection of xenotropic murine leukemia virus-related virus in normal and tumor tissue of patients from the southern United States with prostate cancer is dependent on specific polymerase chain reaction conditions.. J Infect Dis.

[4] Schlaberg R, Choe DJ, Brown KR, Thaker HM, Singh IR (2009). XMRV is present in malignant prostatic epithelium and is associated with prostate cancer, especially high-grade tumors.. Proc Natl Acad Sci U S A.

[5] Cohen J (2011). Retrovirology. More negative data for link between mouse virus and human disease.. Science.

[6] Erlwein O, Kaye S, McClure MO, Weber J, Wills G (2010). Failure to detect the novel retrovirus XMRV in chronic fatigue syndrome.. PLoS One.

[7] Erlwein O, Robinson MJ, Dustan S, Weber J, Kaye S (2011). DNA extraction columns contaminated with murine sequences.. PLoS One.

[8] Erlwein O, Robinson MJ, Kaye S, Wills G, Izui S (2011). Investigation into the presence of and serological response to XMRV in CFS patients.. PLoS One.

[9] Hohn O, Krause H, Barbarotto P, Niederstadt L, Beimforde N (2009). Lack of evidence for xenotropic murine leukemia virus-related virus(XMRV) in German prostate cancer patients.. Retrovirology.

[10] Hohn O, Strohschein K, Brandt AU, Seeher S, Klein S (2011). No evidence for XMRV in German CFS and MS patients with fatigue despite the ability of the virus to infect human blood cells in vitro.. PLoS One.

[11] Hue S, Gray ER, Gall A, Katzourakis A, Tan CP (2011). Disease-associated XMRV sequences are consistent with laboratory contamination.. Retrovirology.

[12] Knox K, Carrigan D, Simmons G, Teque F, Zhou Y (2011). No evidence of murine-like gammaretroviruses in CFS patients previously identified as XMRV-infected.. Science.

[13] Oakes B, Tai AK, Cingoz O, Henefield MH, Levine S (2011). Contamination of human DNA samples with mouse DNA can lead to false detection of XMRV-like sequences.. Retrovirology.

[14] Paprotka T, Delviks-Frankenberry KA, Cingoz O, Martinez A, Kung HJ (2011). Recombinant origin of the retrovirus XMRV.. Science.

[15] Robinson MJ, Erlwein OW, Kaye S, Weber J, Cingoz O (2011). Mouse DNA contamination in human tissue tested for XMRV.. Retrovirology.

[16] Sakuma T, Hue S, Squillace KA, Tonne JM, Blackburn PR (2011). No evidence of XMRV in prostate cancer cohorts in the Midwestern United States.. Retrovirology.

[17] Sato E, Furuta RA, Miyazawa T (2011). An endogenous murine leukemia viral genome contaminant in a commercial RT-PCR kit is amplified using standard primers for XMRV.. Retrovirology.

[18] van Kuppeveld FJ, Jong AS, Lanke KH, Verhaegh GW, Melchers WJ (2010). Prevalence of xenotropic murine leukaemia virus-related virus in patients with chronic fatigue syndrome in the Netherlands: retrospective analysis of samples from an established cohort.. Bmj.

[19] Urisman A, Molinaro RJ, Fischer N, Plummer SJ, Casey G (2006). Identification of a novel Gammaretrovirus in prostate tumors of patients homozygous for R462Q RNASEL variant.. PLoS Pathog.

[20] Silverman RH, Klein EA, Weight CJ, Nguyen C, Das Gupta J (2010). Methods for Detection of XMRV United States Patent Application Publication C12Q 1/70ed. United States..

[21] Menendez-Arias L (2011). Evidence and controversies on the role of XMRV in prostate cancer and chronic fatigue syndrome.. Rev Med Virol.

[22] Furuta RA, Miyazawa T, Sugiyama T, Kuratsune H, Ikeda Y (2010). No association of xenotropic murine leukemia virus-related virus with prostate cancer or chronic fatigue syndrome in Japan.. Retrovirology.

[23] Trottier G, Fleshner NE (2010). Words of wisdom. Re: XMRV is present in malignant prostate epithelium and is associated with prostate cancer, especially high-grade tumors.. Eur Urol.

[24] Aloia AL, Sfanos KS, Isaacs WB, Zheng Q, Maldarelli F (2010). XMRV: a new virus in prostate cancer?. Cancer Res.

[25] Arnold RS, Makarova NV, Osunkoya AO, Suppiah S, Scott TA (2010). XMRV infection in patients with prostate cancer: novel serologic assay and correlation with PCR and FISH.. Urology.

[26] Switzer WM, Jia H, Zheng H, Tang S, Heneine W (2011). No Association of Xenotropic Murine Leukemia Virus-Related Virus with Prostate Cancer.. PLoS One.

[27] Fischer N, Hellwinkel O, Schulz C, Chun FK, Huland H (2008). Prevalence of human gammaretrovirus XMRV in sporadic prostate cancer.. J Clin Virol.

[28] Groom HC, Boucherit VC, Makinson K, Randal E, Baptista S (2010). Absence of xenotropic murine leukaemia virus-related virus in UK patients with chronic fatigue syndrome.. Retrovirology.

[29] Smith RA (2011). Contamination of clinical specimens with MLV-encoding nucleic acids: implications for XMRV and other candidate human retroviruses.. Retrovirology.

[30] Switzer WM, Jia H, Hohn O, Zheng H, Tang S (2010). Absence of evidence of xenotropic murine leukemia virus-related virus infection in persons with chronic fatigue syndrome and healthy controls in the United States.. Retrovirology.

[31] Verhaegh GW, de Jong AS, Smit FP, Jannink SA, Melchers WJ (2011). Prevalence of human xenotropic murine leukemia virus-related gammaretrovirus (XMRV) in Dutch prostate cancer patients.. Prostate.

[32] Alberts B (2011). Editorial expression of concern.. Science.

[33] Tuke PW, Tettmar KI, Tamuri A, Stoye JP, Tedder RS (2011). PCR master mixes harbour murine DNA sequences.. Caveat emptor! PLoS One.

[34] Dong B, Kim S, Hong S, Das Gupta J, Malathi K (2007). An infectious retrovirus susceptible to an IFN antiviral pathway from human prostate tumors.. Proc Natl Acad Sci U S A.

[35] Garson JA, Kellam P, Towers GJ (2011). Analysis of XMRV integration sites from human prostate cancer tissues suggests PCR contamination rather than genuine human infection.. Retrovirology.

[36] Stieler KSC, Lavanya M, Aepfelbacher M, Stocking C, Fischer N (2010). Host range and cellular tropism of the human exogenous gammaretrovirus XMRV..

[37] Simon R, Mirlacher M, Sauter G (2004). Tissue microarrays.. Biotechniques.

[38] Groom HC, Yap MW, Galao RP, Neil SJ, Bishop KN (2010). Susceptibility of xenotropic murine leukemia virus-related virus (XMRV) to retroviral restriction factors.. Proc Natl Acad Sci U S A.

[39] Paprotka T, Venkatachari NJ, Chaipan C, Burdick R, Delviks-Frankenberry KA (2010). Inhibition of xenotropic murine leukemia virus-related virus by APOBEC3 proteins and antiviral drugs.. J Virol.

[40] Rodriguez JJ, Goff SP (2010). Xenotropic murine leukemia virus-related virus establishes an efficient spreading infection and exhibits enhanced transcriptional activity in prostate carcinoma cells.. J Virol.

[41] Stieler K, Fischer N (2010). Apobec 3G efficiently reduces infectivity of the human exogenous gammaretrovirus XMRV.. PLoS One.

[42] Baraniuk JN (2010). Xenotropic murine leukemia virus-related virus in chronic fatigue syndrome and prostate cancer.. Curr Allergy Asthma Rep.

[43] Shin CH, Bateman L, Schlaberg R, Bunker AM, Leonard CJ (2011). Absence of XMRV and other MLV-related viruses in patients with Chronic Fatigue Syndrome.. J Virol.

[44] Chaipan C, Dilley KA, Paprotka T, Delviks-Frankenberry KA, Venkatachari NJ (2011). Severe restriction of xenotropic murine leukemia virus-related virus replication and spread in cultured human peripheral blood mononuclear cells.. J Virol.

