# Supplementary figures and images for: No Detection of XMRV in Blood Samples and Tissue Sections from Prostate Cancer Patients in Northern Europe
Source: PLoS One. 2011 Oct 12;6(10):e25592. doi: 10.1371/journal.pone.0025592 (PMC3192048; doi:10.1371/journal.pone.0025592)

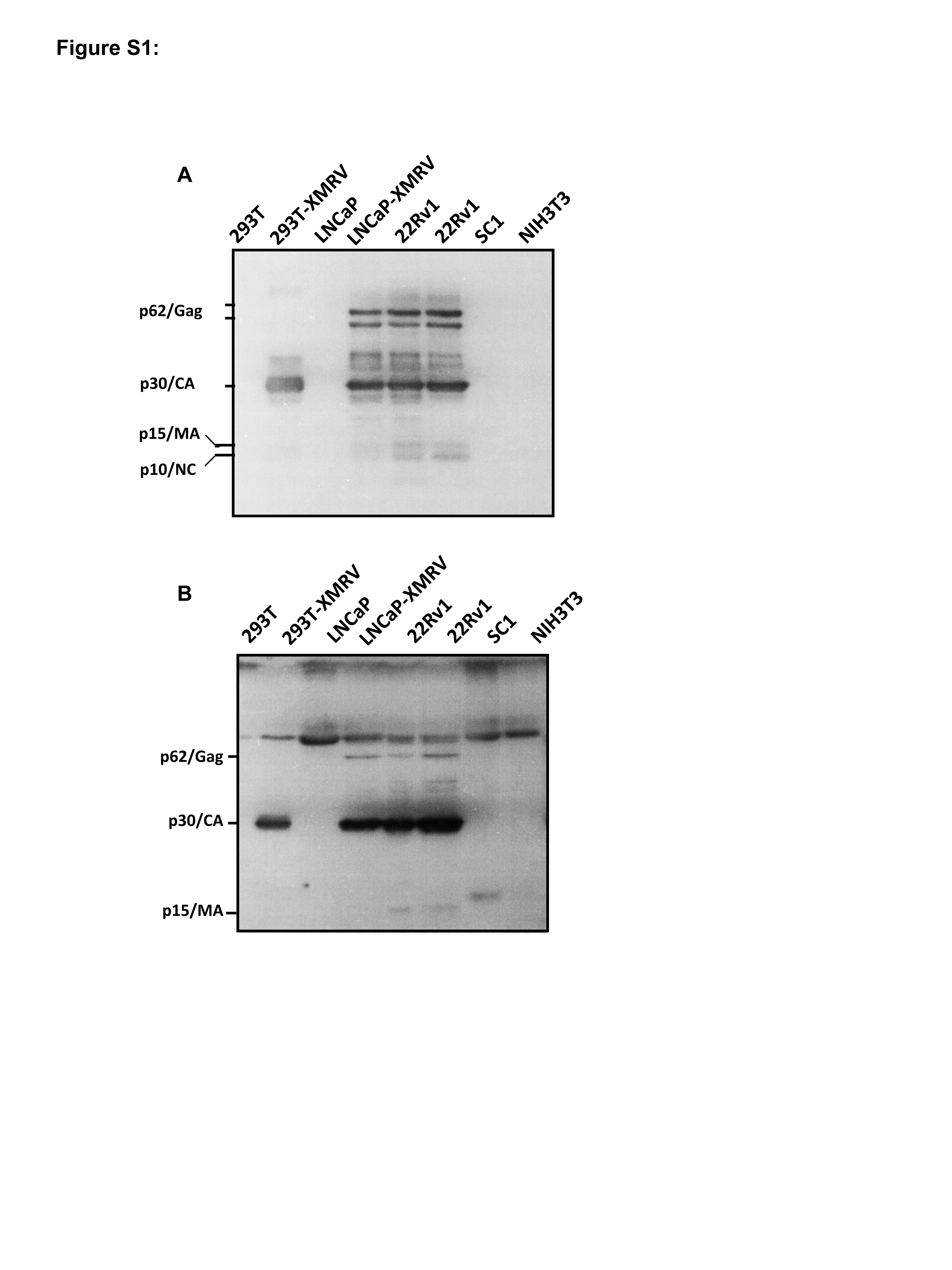

Supplement: Figure S1 — Western Blot analysis of XMRV negative (293T; LNCaP), XMRV positive human cell lines (22Rv1), chronically infected human cell lines (293T-XMRV; LNCaP-XMRV) as well as mouse cell lines (inbred NIH3T3 and feral mouse cells SC1) using rabbit polyclonal α-gag k121 serum (A) or rabbit polyclonal α-XMRV serum [4] (B) for detection. (TIF) [file pone.0025592.s001.tif]

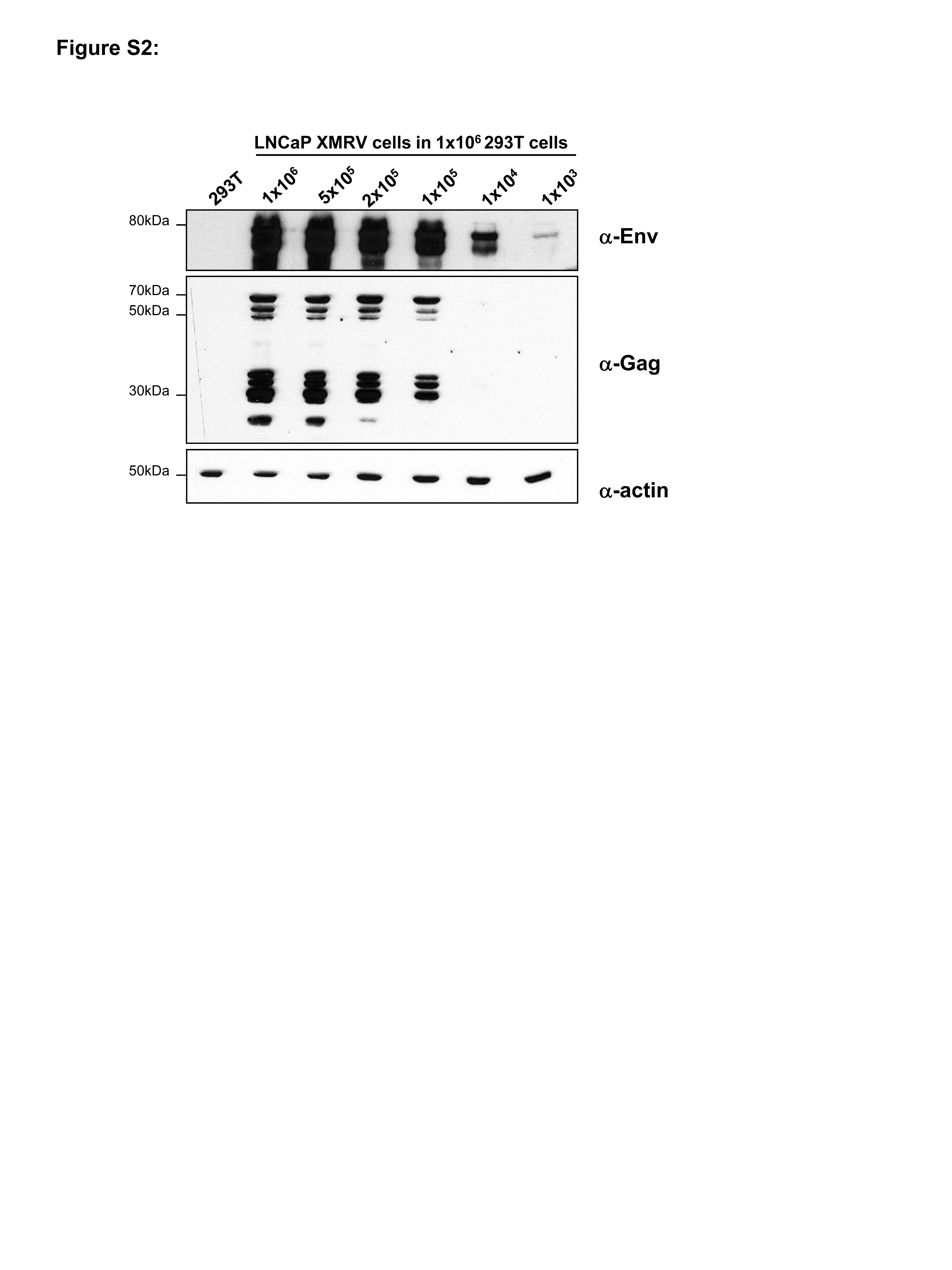

Supplement: Figure S2 — Western Blot analysis of diluting amounts of chronically XMRV infected LNCaP cells mixed with non infected 293T cells. 25 µg total protein lysate was loaded per lane. Blots were immunoblotted using goat-anti env serum and rabbit-anti gag k121 serum. To ensure equal protein amounts loaded per lane the blot was reprobed with anti-actin monoclonal antibody. (TIF) [file pone.0025592.s002.tif]

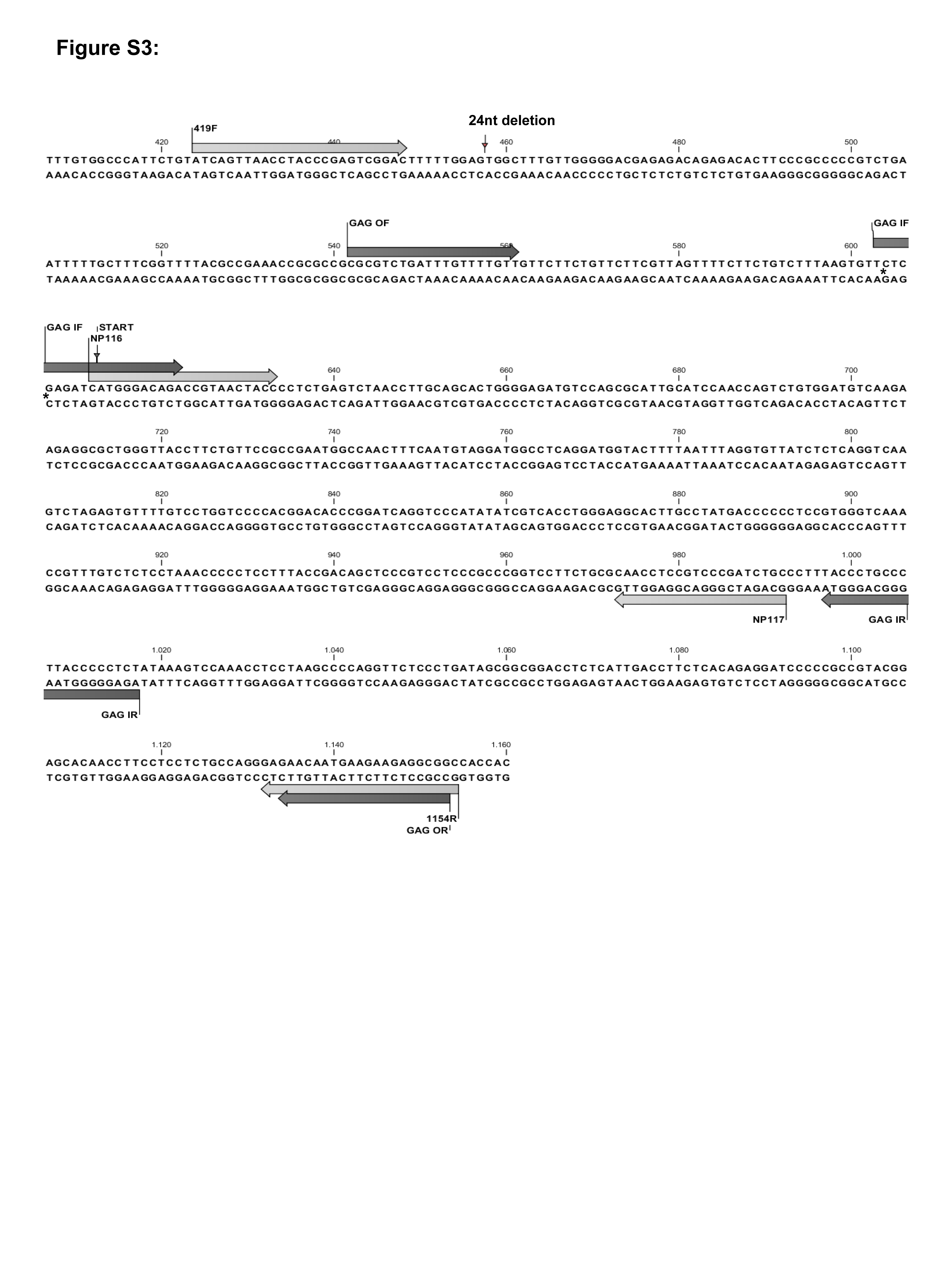

Supplement: Figure S3 — XMRV VP62 Gag sequence 407-1160 (GI:89889045). Primers are indicated as arrows, GAG-O/I dark grey, 419F/1154R and NP116/NP117 light grey. Sequence variability between XMRV and MLV related sequences located in the indicated primer sequences are labeled with a star (*). (TIF) [file pone.0025592.s003.tif]

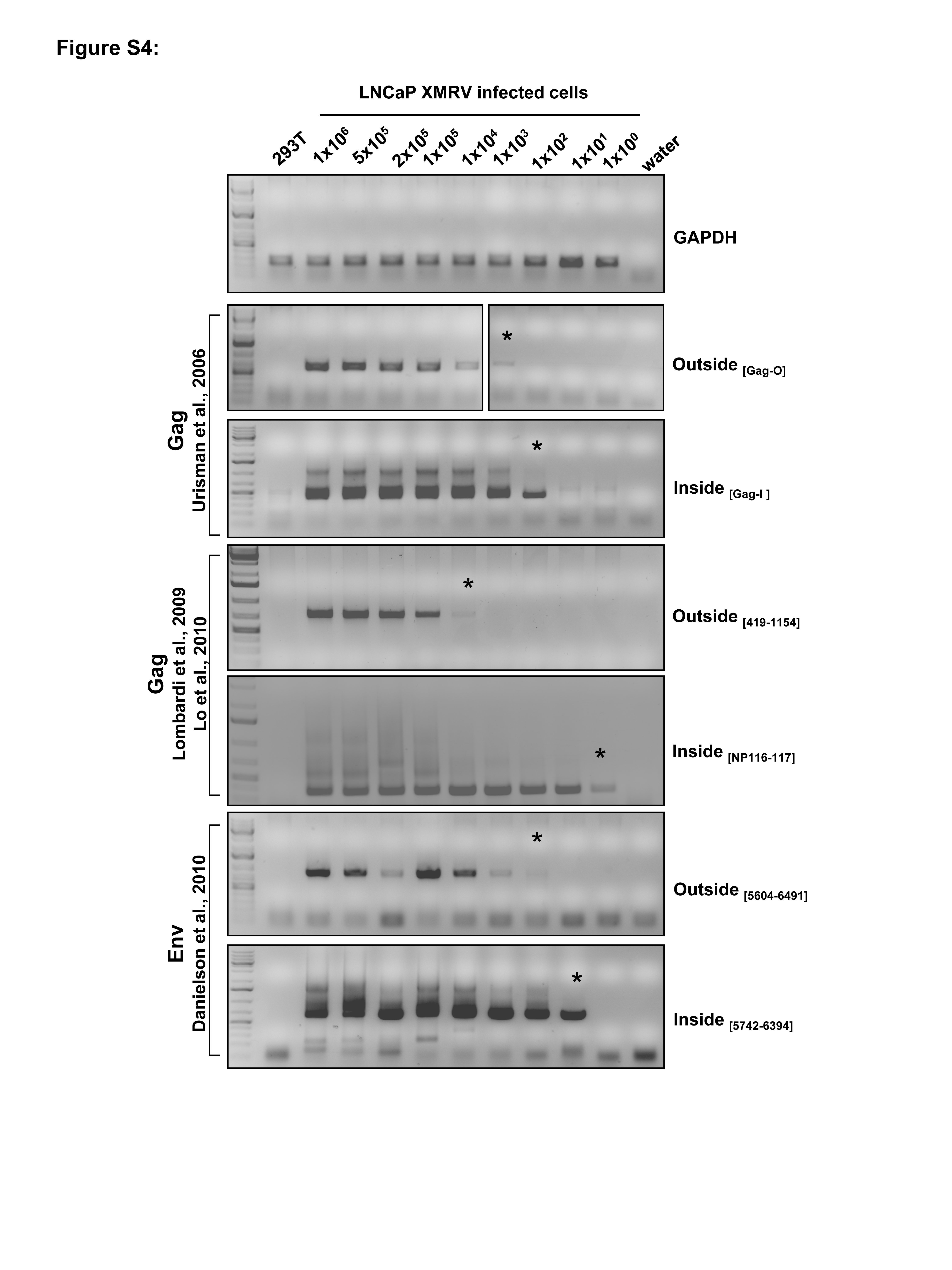

Supplement: Figure S4 — Genomic DNA was isolated from 1×10^6^ cells (indicated number of chronically XMRV infected LNCaP cells mixed with non infected 293T cells in 10 fold dilutions of infected cells in non infected cells). Nested PCR was performed using the oligos GAG-O and GAG-I [19], 419F/1154R and NP116/NP117 [1] as well as env primers 5604F/6491R and 5742F/6394R [3]. The highest dilution still showing XMRV specific amplification products in labelled with an *. (TIF) [file pone.0025592.s004.tif]
